# Comparative venom-gland transcriptomics and venom proteomics of four Sidewinder Rattlesnake (*Crotalus cerastes*) lineages reveal little differential expression despite individual variation

**DOI:** 10.1038/s41598-018-33943-5

**Published:** 2018-10-19

**Authors:** Erich P. Hofmann, Rhett M. Rautsaw, Jason L. Strickland, Matthew L. Holding, Michael P. Hogan, Andrew J. Mason, Darin R. Rokyta, Christopher L. Parkinson

**Affiliations:** 1 Clemson University, Department of Biological Sciences, Clemson, SC 29634 USA; 2 University of Central Florida, Department of Biology, Orlando, FL 32816 USA; 3 Florida State University, Department of Biological Science, Tallahassee, FL 32306 USA; 4 Clemson University, Department of Forestry and Environmental Conservation, Clemson, SC 29634 USA

## Abstract

Changes in gene expression can rapidly influence adaptive traits in the early stages of lineage diversification. Venom is an adaptive trait comprised of numerous toxins used for prey capture and defense. Snake venoms can vary widely between conspecific populations, but the influence of lineage diversification on such compositional differences are unknown. To explore venom differentiation in the early stages of lineage diversification, we used RNA-seq and mass spectrometry to characterize Sidewinder Rattlesnake (*Crotalus cerastes*) venom. We generated the first venom-gland transcriptomes and complementary venom proteomes for eight individuals collected across the United States and tested for expression differences across life history traits and between subspecific, mitochondrial, and phylotranscriptomic hypotheses. Sidewinder venom was comprised primarily of hemorrhagic toxins, with few cases of differential expression attributable to life history or lineage hypotheses. However, phylotranscriptomic lineage comparisons more than doubled instances of significant expression differences compared to all other factors. Nevertheless, only 6.4% of toxins were differentially expressed overall, suggesting that shallow divergence has not led to major changes in Sidewinder venom composition. Our results demonstrate the need for consensus venom-gland transcriptomes based on multiple individuals and highlight the potential for discrepancies in differential expression between different phylogenetic hypotheses.

## Introduction

Lineage diversification allows for evolutionary processes such as selection to act independently upon a trait across different clades. For traits tightly linked to an organism’s survival, adaptation within a lineage can occur rapidly through the evolution of gene-expression patterns (*i.e*. regulatory evolution), where few changes in the regulatory machinery yield large, cascading changes in the expressed phenotype^1,2^. Regulatory evolution contrasts with the evolution of coding sequences in focal genes, which is reliant on the comparatively slow process of mutation in coding gene exons^3^. Notably, variation in gene-expression patterns has contributed to adaptation in many species and may be a key source of variability among populations in the early stages of lineage diversification^1^. However, as many ecologically-important traits are polygenic and involve complex pathways, they can be difficult to track genetically. Traits with a tractable genetic basis and high ecological importance can therefore permit investigations of the mode, tempo, and correlates of adaptive regulatory evolution in young lineages^2^.

Venomous animals provide exceptional systems to investigate the role of regulatory evolution on adaptive traits in the early stages of lineage diversification^4^. Venom is a polygenic trait that has evolved multiple times across the tree of life, where it serves in prey capture and predator defense^4,5^. Unlike many polygenic traits^2,6^, venom is the result of a relatively direct pathway from the transcription of toxin genes to the translation of toxin proteins, which are then stored as a protein mixture prior to use^7,8^. Therefore, by combining venom-gland transcriptomics and venom proteomics, we can accurately map the progression from genotype to phenotype in this adaptive trait^4^.

Prior proteomic, enzymatic, and transcriptomic studies have found significant variation in the composition and expression of snake venom across all levels of biological organization, including between populations of the same species^9^. This extensive variation is both biologically significant and medically important: for example, venomous snake families (*i.e*. Viperidae, Elapidae) often share similar toxins based on phylogenetic relatedness^10^. However, within rattlesnakes (*Crotalus* and *Sistrurus*), little evidence exists for phylogenetic signal in patterns of venom expression between species, indicating rapid local adaptation in allopatry^10–13^. Most studies of venom variation in rattlesnakes focus on populations of single species, where geographic variation is commonly detected and associated with variable diets^14,15^, local environments^16^, or interactions with coevolving prey^16,17^. These patterns are largely attributed to differential gene expression^17–20^. However, few intraspecific studies have explored the role of differential gene expression in driving venom variation where there is phylogenetic structure within a species across its range. At this level, if we find patterns of differential toxin expression, then it could be due to general accumulation of change as the lineages diversify in addition to local adaptation to prey^17,19,21^.

**Figure 1:**
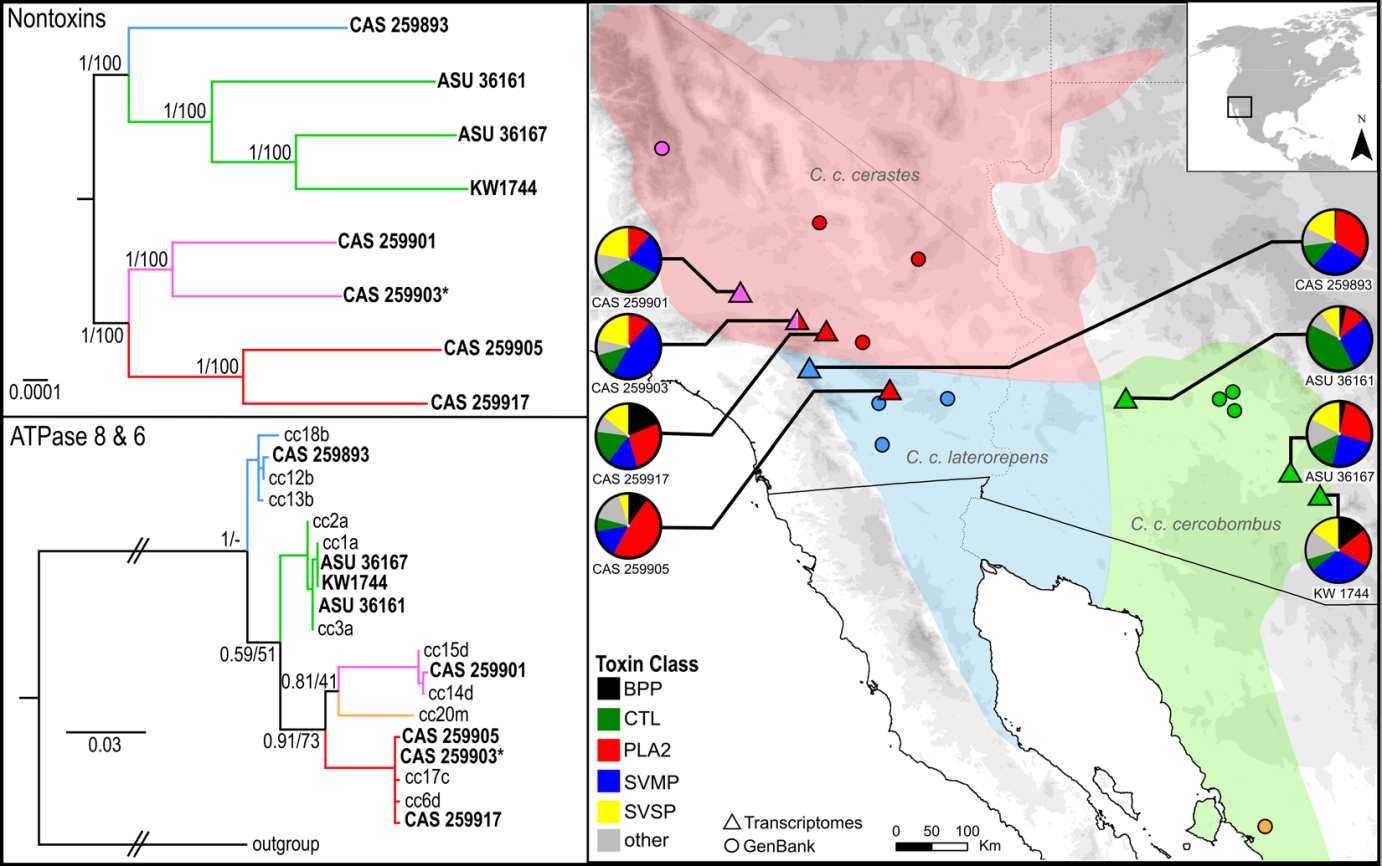
Sampling and approximate distribution of *Crotalus cerastes* by subspecies. Phylogeny in upper panel based on 1,508 nontoxin loci and 1,401,828 bp, rooted at the midpoint. Phylogeny in the lower panel based on the mtDNA loci ATPase 8 and 6, using samples from GenBank (Douglas *et al*.^28^) and transcriptomes generated herein. Sample colors correspond to recovered clade, not assignment based on proposed subspecies ranges. Note the difference in the position of CAS 259903 between trees. Pie charts correspond to toxin gene expression in each sample, showing the five most highly expressed toxin families. Maps were created using ArcGIS 10.5.1 (ESRI Inc., USA. http://www.esri.com) using freely available spatial data (www.diva-gis.org/Data), then annotated in Inkscape 0.92 (https://inkscape.org); species ranges were approximated based on^83^.

Here, we attempt to understand the effect of shallow lineage divergence on venom through quantification of the transcriptomes and proteomes of distinct lineages of Sidewinder Rattlesnakes (*Crotalus cerastes*). Sidewinders are small rattlesnakes native to the warm deserts of the southwestern United States and northwestern Mexico, easily recognized by their conspicuous, raised supraoculars and specialized side-winding locomotion^22,23^. Previous studies have noted some differences in venom enzymatic activity between populations, but the proteomic basis for this activity is unknown^24,25^. Three subspecies are currently recognized within *Crotalus cerastes*^26^: *C. c. cerastes* found in the Mojave Desert, *C. c. cercobombus* in the Sonoran Desert, and *C. c. laterorepens* in the Colorado Desert (Fig. 1). However, these subspecific assignments are not consistent with phylogenetic lineages. Mitochondrial phylogenetic analyses of *C. cerastes* by Pece^27^ and Douglas *et al*.^28^ recovered four shallow lineages in the United States, corresponding to the North and South Mojave, Colorado, and Sonoran Deserts; Douglas *et al*.^28^ recovered a fifth lineage from the southern portion of the Sonoran Desert in Mexico. It is well-documented that phylogenies built solely on mitochondrial data may not accurately represent the true evolutionary history or relationships of the study taxa, as the mitochondrial genome is rapidly evolving and matrilineal^29–34^. Therefore, it is important to verify these lineage assignments with larger genomic datasets. Sidewinder subspecies and mitochondrial lineages are suitable as initial hypotheses to test for differential venom expression, but the considerable number of nontoxin genes captured by venom-gland transcriptomics allows for a more robust investigation of the evolutionary relationships of these populations.

Based on previous studies suggesting variable enzymatic activity within *Crotalus cerastes* venom^24,25,35^ and the well-documented intraspecific variation in venom from other co-distributed Crotaline taxa^36–38^, we hypothesize that lineage diversification has led to venom divergence within the species and predict this divergence will correspond to lineage, subspecies, and/or life history. To test this hypothesis, we describe the first venom-gland transcriptomes and corresponding venom proteomes of eight *Crotalus cerastes*, including samples assignable to all subspecies and mitochondrial lineages known from the southwestern United States. We test whether life history traits (sex or age) or lineage assignments (subspecies, mitochondrial or phylotranscriptomic lineages) best explain differences in toxin expression, and subsequently explore the influence of lineage diversification on differential toxin expression.

## Methods

### Specimen, venom, and venom gland collection

We collected representative samples from each of the recognized subspecies of *C. cerastes*. In total, eight snakes (six males, two females) were collected between October 2012 and July 2014 in California and Arizona (Fig. 1; Table 1). Snout-to-vent lengths (SVL) ranged from 30.9–48.4 cm (43.12 ± 7.12 cm) for males and 37.7–40.8 cm (39.2 ± 1.55 cm) for females. Snakes with SVL less than 34 cm (males) or 38 cm (females) were consider juveniles^39,40^. We collected venom from the snakes, then four days later collected venom glands from each snake to maximize transcription of venom genes^41^. Venom was collected by either allowing the snakes to bite onto a parafilm-sealed sterile collection cup or by electrostimulation^42^, then dehydrated and stored at −80 °C until analyzed. Four days after venom extraction, snakes were euthanized with a single-step sodium pentobarbital (100 mg/kg) injection following standard approved AVMA guidelines^43^, and both the left and right venom glands of each snake were excised. The excised venom glands were immediately transferred to RNAlater following dissection and stored briefly at 4 °C prior to long term storage at −80 °C. Snakes were handled and collected under the following permits and Animal Care and Use Protocols: State of Arizona Game and Fish Department (SP673390), California Natural Resources Agency Department of Fish and Wildlife (SC-12985), and University of Central Florida IACUC (13–17 W).

**Table 1:** Eight *Crotalus cerastes* used in this study and associated metadata.

Museum ID	Field ID	Subspecies	mtDNA Lineage	Nontoxin Lineage	Sex	SVL (cm)	Read Pairs	Merged Reads	SRA Accession
CAS 259901	CLP2065	*cerastes*	N Mojave	N Mojave	M	47.8	13,285,200	11,730,533	SRR6768683
CAS 259903	CLP2068	*cerastes*	S Mojave	N Mojave	M	39.1	11,295,627	10,012,070	SRR6768684
CAS 259917	CLP2109	*cerastes*	S Mojave	S Mojave	F	40.8	11,482,030	9,944,019	SRR6768687
CAS 259905	CLP2071	*laterorepens*	S Mojave	S Mojave	F	37.7	14,205,702	12,049,288	SRR6768685
CAS 259893	CLP2057	*laterorepens*	Colorado	Colorado	M	30.9	12,801,696	11,315,608	SRR6768682
ASU 36161	CLP2105	*cercobombus*	Sonora	Sonora	M	48.4	13,508,336	11,041,594	SRR6768686
ASU 36167	CLP2137	*cercobombus*	Sonora	Sonora	M	45.0	10,860,478	9,634,955	SRR6768688
—	KW1744	*cercobombus*	Sonora	Sonora	M	43.5	17,607,533	15,388,424	SRR6768689

### RNA extraction and sequencing

We isolated RNA via a standard TRIzol extraction following Rokyta *et al*.^20,44,45^. Briefly, venom glands were finely diced and placed in TRIzol solution (Invitrogen). The mixture was homogenized and transferred to a phase lock heavy gel tube (5Prime). Once cells were lysed, total RNA was isolated using chloroform and purified via isopropyl alcohol and ethanol precipitation. RNA quantification was performed using a Qubit RNA BroadRange kit. RNA quality was checked using a Bioanalyzer 2100 with an RNA 6000 Pico Kit (Agilent Technologies) or Agilent TapeStation 2200 with a RNA ScreenTape to ensure sufficient quantity and quality RNA for library preparation and sequencing.

We produced cDNA libraries from isolated mRNA using magnetic bead isolation of mRNA followed by cDNA synthesis and PCR amplification. First, we isolated mRNA using the NEBNext Poly(A) mRNA Magnetic Isolation Module (NEB #E7490S) with equal amounts of mRNA from each of the left and right venom glands. Following bead isolation and cleanup, cDNA libraries were prepared from isolated mRNA using a NEB Next Ultra RNA Library Prep Kit for Illumina (NEB #E7530) following manufacturer’s recommendations. We used a fragmentation time of 13 minutes, 30 seconds to achieve a target mean fragment size of 400 bp, and 14 PCR cycles for amplification of double stranded cDNA libraries. Library yield and quality was quantified on a Bioanalyzer 2100. The total amplifiable concentration of cDNA in each library was then determined using KAPA qPCR at the Florida State University Molecular Cloning Facility. Equal concentrations of samples were then pooled in groups of 12 samples for sequence. The final concentration and quality of the pooled libraries samples was then assessed on the Bioanalyzer and via KAPA qPCR. Pooled libraries were sequenced with 150 base pair (bp) paired-end reads on an Illumina HiSeq2500 platform at the Florida State University College of Medicine Translational Science Laboratory (Tallahassee, FL, USA).

### Transcriptome assembly and annotation

We cleaned and assembled the raw reads using a combination of custom python scripts and established software. The raw 150 bp paired-end reads were checked for potential cross-contamination, caused by mis-allocation of reads during demultiplexing, using custom python scripts. The scripts first obtain counts of all 57-mers in the raw reads using Jellyfish v. 2.2.6^46^. The scripts then obtain all pairwise comparisons of these k-mer counts for samples in a given lane, and removes reads that were 25% constructed of these k-mers. Cleaned reads were then trimmed to keep base calls with a phred score of 5 or greater using Trim Galore! v. 0.4.4 and merged using PEAR v. 0.9.10^47^. The trimmed, merged reads were *de novo* assembled using two different methods: Extender^44^ and SeqMan NGen (using the Lasergene DNAStar software package; Madison, WI: https://www.dnastar.com/t-nextgen-seqman-ngen.aspx). Extender assemblies used 1000 merged reads as seeds, extending the seeds based on exact overlaps of 120 base pairs. This method has efficiently assembled toxin sequences in a variety of snake taxa, including other *Crotalus* species^19,20,44,45,48–50^. *De novo* NGen assemblies were performed with default settings in NGen v. 14.

Assembled contigs from both Extender and NGen were annotated via blastx searches against the UniProt animal venom proteins and toxins database (http://www.uniprot.org/program/Toxins) with a minimum e-value of 10^−4^. Both toxins and nontoxins (*e.g*. housekeeping genes) were annotated by clustering sequences using cd-hit-est^51^ to a known database of previously annotated snake toxins and *C. horridus* nontoxins^48^. Sequences and associated signal peptides with a match percentage of 80 were automatically annotated. The remaining toxin contigs were manually annotated by comparing the sequences to the blastx results. We then combined annotated sequences from both assembly methods, removed duplicates, and screened for chimeric sequences by aligning merged reads to the annotated toxins using BWA-MEM^52^ and removing reads with any mismatches via gaps or nucleotide differences. Transcripts with zero coverage at any base were automatically removed, and transcripts with more than 10-fold coverage differentials across their length were removed if they showed signs of chimeric transcripts (multimodal coverage distributions). The remaining transcripts were clustered with a sequence identity threshold of 98% using cd-hit to reduce redundancy of repeat transcripts and cluster allelic variation at single loci^51^. Finally, the *de novo*-assembled transcriptomes of each individual were combined and clustered using cd-hit with a sequence identity threshold of 98% to produce a species consensus transcriptome.

### Phylogenetic analysis

We assigned our samples to known phylogeographic lineages first using previously published sequence data as well as using the annotated nontoxins recovered from the transcriptomes. First, we obtained partial sequences (665 bp) of the mitochondrial loci ATPase 8 and 6 from our samples by mapping each individual’s merged reads to a reference sequence obtained from GenBank using Bowtie 2 (DQ493803, see below)^53^. These sequences were aligned with eleven sequences obtained from GenBank corresponding to the five mitochondrial lineages of *Crotalus cerastes* recovered by Douglas *et al*.^28^ (DQ493803–DQ493813), the most complete molecular sampling of the species available to date (Fig. 1). An additional sequence assigned to *C. helleri* (AF462375; taxonomy following Davis *et al*.^54^) was included as an outgroup. Our goal was not to explore evolutionary relationships with only a small dataset of mtDNA, rather to cluster our samples with the appropriate phylogeographic clades corresponding to the mitochondrial lineages recovered by Douglas *et al*.^28^.

We also used a subset of the automatically annotated nontoxins for a more robust phylotranscriptomic analysis. First, merged reads for each individual were mapped to the consensus transcriptome using BWA-MEM^52^ and reads with more than three mismatches—via gaps or nucleotide differences—were removed. Picard (http://broadinstitute.github.io/picard/) was used to sort and index the aligned reads prior to using bedtools to calculate the coverage of each site within each transcript. Using a custom R script, any transcript which had zero coverage for any site in a transcript was marked as absent. Nontoxins which were present across all eight of our individuals were then pulled and used to re-map the merged reads with BWA-MEM again. Alleles were phased using a combination of samtools, Picard, and GATK (following^55^) and concatenated using Sequence Matrix^56^. The final dataset consisted of 1,508 nontoxin loci, and 1,401,828 bp.

We used both maximum likelihood and Bayesian phylogenetic approaches to recover lineage assignments for our samples. We carried out maximum likelihood (ML) analyses in RAxML v. 8.2.4^57^ with 1000 bootstrap pseudoreplicates under the default GTR + G substitution model. Bayesian inference (BI) was performed using MrBayes 3.5.2^58,59^. For the Bayesian analysis, ATPase 8 and 6 were partitioned by codon, and we used PartitionFinder v. 1.1.1^60^ to determine best fit models of nucleotide substitution, with a greedy search algorithm and the model search restricted to those implementable in MrBayes. The GTR + G model was chosen as the best model for all codons except ATPase 6 codons 2 (GTR + I) and 3 (HKY). For the nontoxin dataset, we used the GTR + G model partitioned by locus. The analyses consisted of two parallel runs of four Markov chains (three heated, one cold) run for 100 million generations, sampling every 10,000, with a random starting tree and the first 20% discarded as burnin.

### Venom proteomics

To generate a genotype-phenotype map and verify toxin expression proteomically, we performed quantitative mass spectrometry (qMS) on whole venom samples following Rokyta & Ward^61^ and Ward *et al*.^62^. We first quantified venom protein samples using the Qubit Protein Assay kit with a Qubit 1.0 Fluorometer (Thermo Fisher Scientific). For each sample, we then digested approximately 5 *μ*g of whole venom using the Calbiochem ProteoExtract All-in-One Trypsin Digestion Kit (Merck, Darmstadt, Germany) according to the manufacturer’s instructions and using LC/MS grade solvents, leaving an overall yield of approximately 4.3 *μ*g of digested venom protein after digestion. Samples were dried using a SpeedVac at 25 °C for 1 hour and stored at −20 °C until use. To initiate the mass spectrometry run, the resulting dried and digested tryptic peptides were resuspended in 0.1% formic acid at a final concentration of 250 ng/*μ*L. Three digested *Escherichia coli* proteins-purchased from Abcam at known concentrations and mixed in the specified proportions (1000×) prior to digestion-were used as internal standards: 25 fmol of P00811 (Beta-lactamase ampC), 250 fmol of P31658 (Protein deglycase 1), and 2500 fmol of P31697 (Chaperone protein FimC) per injection. Final sample concentrations were achieved by infusing the internal standard peptide mix into samples. For the LCMS/MS run, a 2 *μ*L aliquot was analyzed using an externally calibrated Thermo Q Exactive HF (high-resolution electrospray tandem mass spectrometer) in conjunction with Dionex UltiMate3000 RSLCnano System. A 2 *μ*L sample was aspirated into a 50 *μ*L loop and loaded onto the trap column (Thermo *μ*-Precolumn 5 mm, with nanoViper tubing 30 *μ*m i.d. ×10 cm). For separation on the analytical column (Acclaim pepmap RSLC 75 *μ*Mx 15 cm nanoviper), the flow rate was set to 300 nl/min. Mobile phase A was composed of 99.9% H2O (EMD Omni Solvent) and 0.1% formic acid, and mobile phase B was composed of 99.9% ACN and 0.1% formic acid. We performed a 60 min linear gradient from 3% to 45% B. The LC eluent was directly nanosprayed into the Q Exactive HF mass spectrometer (Thermo Scientific), and during the chromatographic separation, the Q Exactive HF was operated in a data-dependent mode and under direct control of the Thermo Excalibur 3.1.66 (Thermo Scientific). Resulting MS data were acquired using a data-dependent top-20 method for the Q Exactive HF platform, dynamically choosing the most abundant not-yet-sequenced precursor ions from the survey scans (350–1700).

Sequencing was performed via higher energy collisional dissociation fragmentation with a target value of 10^5^ ions determined with predictive automatic gain control. Full scans (350–1700 m/z) were performed at 60,000 resolution in profile mode. MS2 were acquired in centroid mode at 15,000 resolution. We excluded ions with a single charge, charges more than seven, or unassigned charge. A 15-s dynamic exclusion window was used. All measurements were performed at room temperature, and done with three technical replicates to account for machine-related variability and to facilitate label-free quantification. We searched the resulting raw files with Proteome Discoverer 1.4 using SequestHT as the search engine with custom-generated FASTA databases and percolator as peptide validator. The SequestHT search parameters used were: enzyme name = Trypsin, maximum missed cleavage = 2, minimum peptide length = 6, maximum peptide length = 144, maximum delta Cn = 0.05, precursor mass tolerance = 10 ppm, fragment mass tolerance = 0.2 Da, dynamic modifications, carbamidomethyl + 57.021 Da(C) and oxidation + 15.995 Da(M). Protein identities were validated using Scaffold (version 4.3.4, Proteome Software Inc., Portland, OR, USA) software. We accepted protein identities based on a 1.0% false discovery rate (FDR) using the Scaffold Local FDR algorithm and a minimum of one recognized peptide. We considered a transcript proteomically detected if it was found in at least one of the three replicates per individual. Any protein not detected in all three replicates was excluded from further analyses. From all individuals we also excluded Bradykinin-potentiation peptides due to the potential for extensive proteolytic cleavage^49^, as well as myotoxins due to low quality assignments. Finally, individual toxins that appeared to be ambiguously assigned in all three replicates of a sample were omitted.

To estimate proteomic abundances, we followed Rokyta & Ward^61^ and Ward *et al*.^62^ and calculated separate conversion factors for each of three replicates based on the known concentrations of the *Escherichia coli* control proteins and their observed quantitative values (normalized spectral counts) determined by Scaffold. These conversion factors were calculated by finding the slope of the best fit line of the known control concentrations and the observed normalized spectral counts, with an intercept at the origin. The conversions factors were then used to convert the normalized spectral counts for each venom protein in each replicate to concentrations; final concentrations for each sample were then averaged across each individual’s corresponding replicates. The centered log-ratio (clr) transformation^63^ was applied to all our transcriptome and proteome abundances for these analyses; this transformation preserves rank and is equivalent to a log transformation for linear relationships^49^.

The methods used here are designed to provide information on relative protein abundances, primarily to validate transcriptomic findings. In snakes, the internal standard approach has previously been shown to provide a strong agreement between venom proteins and transcripts across multiple species^49^. Using this approach to determine absolute protein abundances has limitations, however, due to protein-specific properties which can affect downstream abundance calculations. For this reason, all analyses are done in a compositional framework. Prior work has shown that different approaches to quantification do not affect correlation between mRNA and protein levels using the internal standard approach^49^. Although additional proteomic and/or analytical methods for identification and quantification may afford greater detectability of specific toxins (*e.g.* BPPs, myotoxins), these methods introduce other potential biases to quantification. Rather than potentially biasing our results by using different parameters for different proteins, we chose instead to treat all proteins equally using the same parameters across all samples.

### Comparative transcriptome analyses

We measured relative expression of each venom gene by calculating the number of transcripts per million reads (TPM) and compared our individuals with hierarchical clustering. Using RSEM^64^ with default Bowtie 2 alignment settings^53^, merged reads from each individual were mapped to the consensus transcriptome to calculate the transcripts per million reads (TPM). To avoid zero values in the dataset and allow transformations, we used the ‘cmultRepl’ function in the R package zCompositions^65^. We used the R package ‘pheatmap’^66^ to generate a heatmap hierarchically-clustered by expression similarity (natural log-transformed TPM) between individuals in order to visualize differences in the expression of individual toxin transcripts.

To determine if consensus transcripts were potentially absent in any individuals, we aligned the merged reads of each individual to the consensus transcriptome following Rokyta *et al*.^20^ and calculated coverage of each site. Briefly, alignments were carried out using BWA MEM^52^ and reads with more than three mismatches—via gaps or nucleotide differences—were removed. Picard (http://broadinstitute.github.io/picard/) was used to sort and index the aligned reads prior to using bedtools to calculate the coverage of each site within each transcript. Using a custom R script, transcripts with less than 5x coverage for more than 10% of the coding sequence were considered absent from that individual’s transcriptome^20^. This cutoff was not applied to differential expression analyses; all transcripts were considered present in subsequent analyses.

We tested for differential venom gene expression between life history traits, phylogenetic structure, and subspecific assignment of each individual snake (Table 1). We used DESeq2^67^ and edgeR^68^ to test for differential expression—based on RSEM TPM counts for each venom transcript—across five factors: body size (SVL, corresponding to life stage), sex, mitochondrial lineage, nontoxin (*i.e*. phylotranscriptomic) lineage, and historical subspecies assignment. Mitochondrial and nontoxin lineages largely corresponded to the deserts in which the snakes were found, and are therefore named as in Table 1. Pairwise contrasts were made between the discrete factors: *i.e*. different clades (*e.g*. South Mojave vs. North Mojave), subspecies (*e.g. C. c. cerastes* vs. *C. c. cercobombus*), and sex. Seventeen pairwise comparisons were made in total (Supplementary Tables S1 and S2). For analysis with DESeq2, we performed Wald significance tests with a local fit of dispersions for all comparisons except nontoxin lineage comparisons which was able to use a parametric fit of dispersions. Independent filtering was applied at *P* = 0.05 and *P*-values were corrected for false-discovery rate (FDR). For analysis with edgeR, negative binomial generalized linear models were fit for each gene and significance tested with likelihood ratio tests. Similar to DESeq2, *P*-values were corrected for FDR. We used *α* = 0.05 for detecting differentially expressed genes following FDR-correction in both DESeq2 and edgeR analyses. Genes were considered significantly differentially expressed if they were found to be significant in both DESeq2 and edgeR analyses. We also quantified the phylogenetic signal of venom gene expression by calculating Blomberg’s *K*^69^ in the R package ‘picante’^70^ using the nontoxin phylogeny rooted at the midpoint. This method assumes evolution under Brownian motion, and provides a measure of whether the evolution of a trait matches what is expected (*K* = 1), is less similar between closely related taxa than expected (*K* < 1), or is more similar between closely related taxa than expected (*K* > 1)^69^.

Lastly, we sought to detect toxin class-specific variation in gene expression. To this end, we summed TPM counts by toxin class–across paralogous toxins from the same gene family–to account for potential differences in venom not captured by individual transcript analysis. The new counts of toxin class abundance were similarly used to generate hierarchically clustered heatmaps and test for differential expression as described above.

### Accession codes

SRA: SRR6768682–SRR6768689; TSA: GGMJ00000000

## Results

### Phylogenetic analysis

Our phylogenetic analyses recovered incongruent topologies between the mtDNA-only dataset (two loci, 665 bp) and the nontoxin phylotranscriptomic dataset (1,508 loci, ~1.4 million bp). The mtDNA topologies were poorly supported at several nodes (Fig. 1), but clustered our samples with four of the five known mtDNA lineages. The remaining mitochondrial lineage is found only in South Sonora, Mexico, which we were unable to sample as part of this study. The transcriptomically-derived nontoxin topologies were all strongly supported (PP = 1, BS = 100 for all nodes), largely clustering our samples into clades corresponding to the mtDNA assignments. However, one sample, CAS 259903, which was recovered with the South Mojave lineage in the mtDNA analyes, was recovered most closely related to the North Mojave lineage (CAS 259901), distinct from the other two samples assignable to the South Mojave group (CAS 259905, CAS 259917). This was geographically located between the individuals consistently recovered from the North Mojave and South Mojave lineages, suggesting gene flow may be occurring between these lineages.

### The Sidewinder venom-gland transcriptome

**Figure 2:**
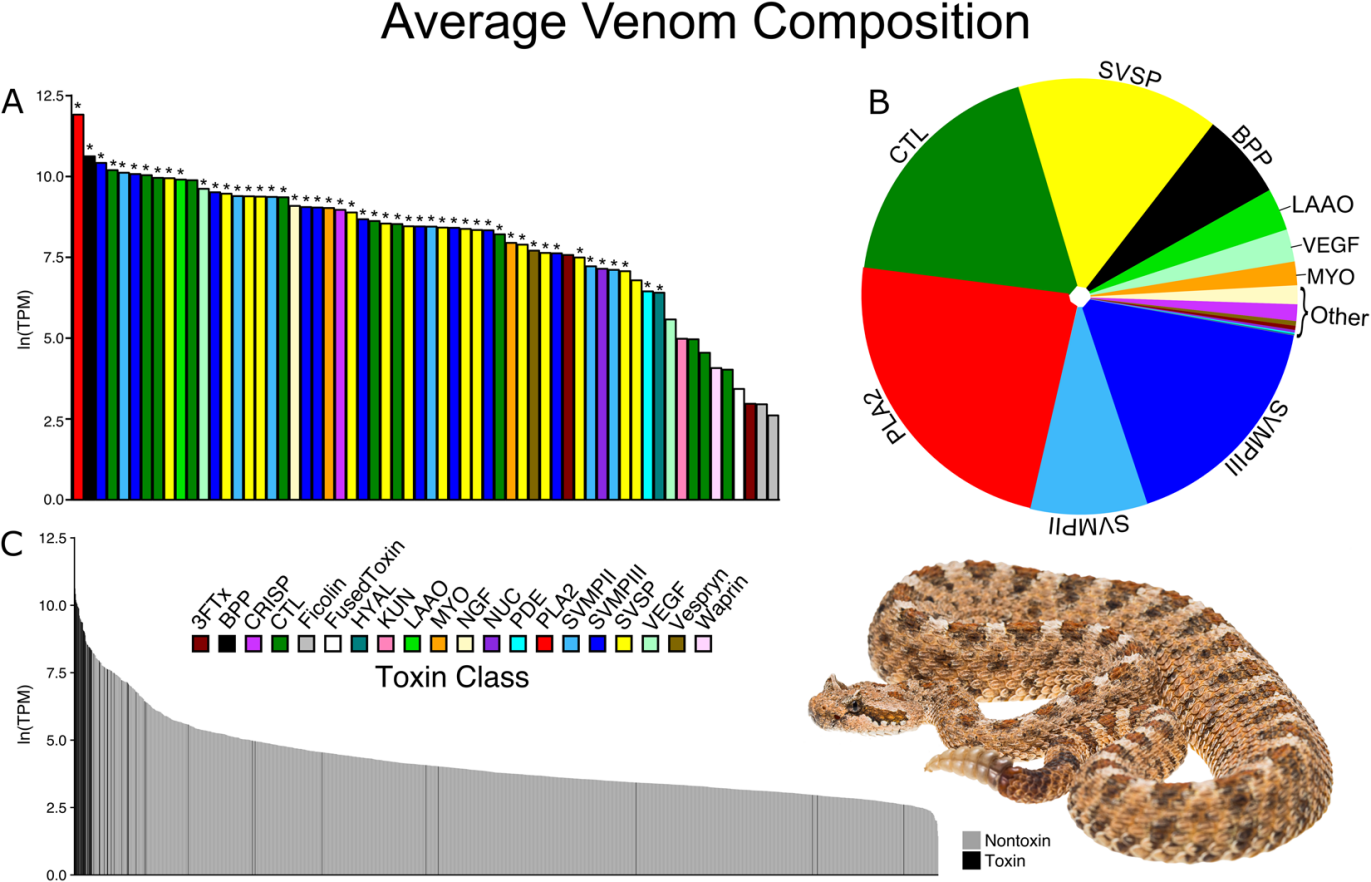
Average expression of the venom-gland transcriptome of *Crotalus cerastes*. (**A**) Expression of each recovered toxin transcript. An asterisk (*) above a toxin indicates it was verified proteomically in at least one individual. (**B**) Proportion of toxin gene expression by class. (**C**) Toxin and nontoxin gene expression in the venom gland. Inset photo: *C. cerastes* by Travis Fisher.

*De novo* assembly and annotation of the venom-gland transcriptome from our eight *C. cerastes* individuals recovered an average of 1,167 unique nontoxin transcripts (range: 909–1,409) and 38 unique toxin transcripts (range: 33–44) per individual. We merged the eight independently assembled transcriptomes to create a consensus venom-gland transcriptome for this species. Our consensus transcriptome consisted of 1,977 unique nontoxin transcripts and 62 unique toxin transcripts (Fig. 2).

Like many other rattlesnakes, *C. cerastes* venom was dominated by snake venom metalloproteinases (SVMP), phospholipase A2 (PLA2), C-type lectins (CTL), snake venom serine proteinases (SVSP), and a bradykinin-protentiating peptide (BPP). Overall, toxins accounted for 63.8% of the transcriptome’s expression based on RSEM-mapped reads across the eight individuals. Five toxin classes made up the majority of *Crotalus cerastes* venom (Fig. 2). SVMPs were the most diverse and abundant toxin class, with a total of 16 unique transcripts (6 SVMPIIs, 10 SVMPIIIs) accounting for 25.8% of toxin-specific expression (Fig. 2). The most highly expressed toxin, a single PLA2, accounted for 23.4% of toxin expression (Fig. 2). The 11 recovered CTLs and 15 SVSPs accounted for 18.3% and 15.1% of the toxin expression, respectively (Fig. 2). Lastly, the second most highly expressed toxin, a single BPP, accounted for 6.4% of toxin expression.

Many less-abundant toxins were present in the venom-gland transcriptome. These included a L-amino-acid oxidase (LAAO), two vascular endothelial growth factors (VEGF), two myotoxins (MYO), a nerve growth factor (NGF), a cysteine-rich secretory protein (CRISP), two 3-fingered toxins (3FTx), a vespryn, a nucleotidase (NUC), and a phosphodiesterase (PDE). The least abundant toxin genes in the venom-gland transcriptome were those that may only have indirect toxic function or no toxic function at all^71^. These include a hyaluronidase (HYAL), a Kunitz-type proteinase inhibitor (KUN), a waprin, two ficolin, and a fused toxin.

### Comparative transcriptome analysis

**Figure 3:**
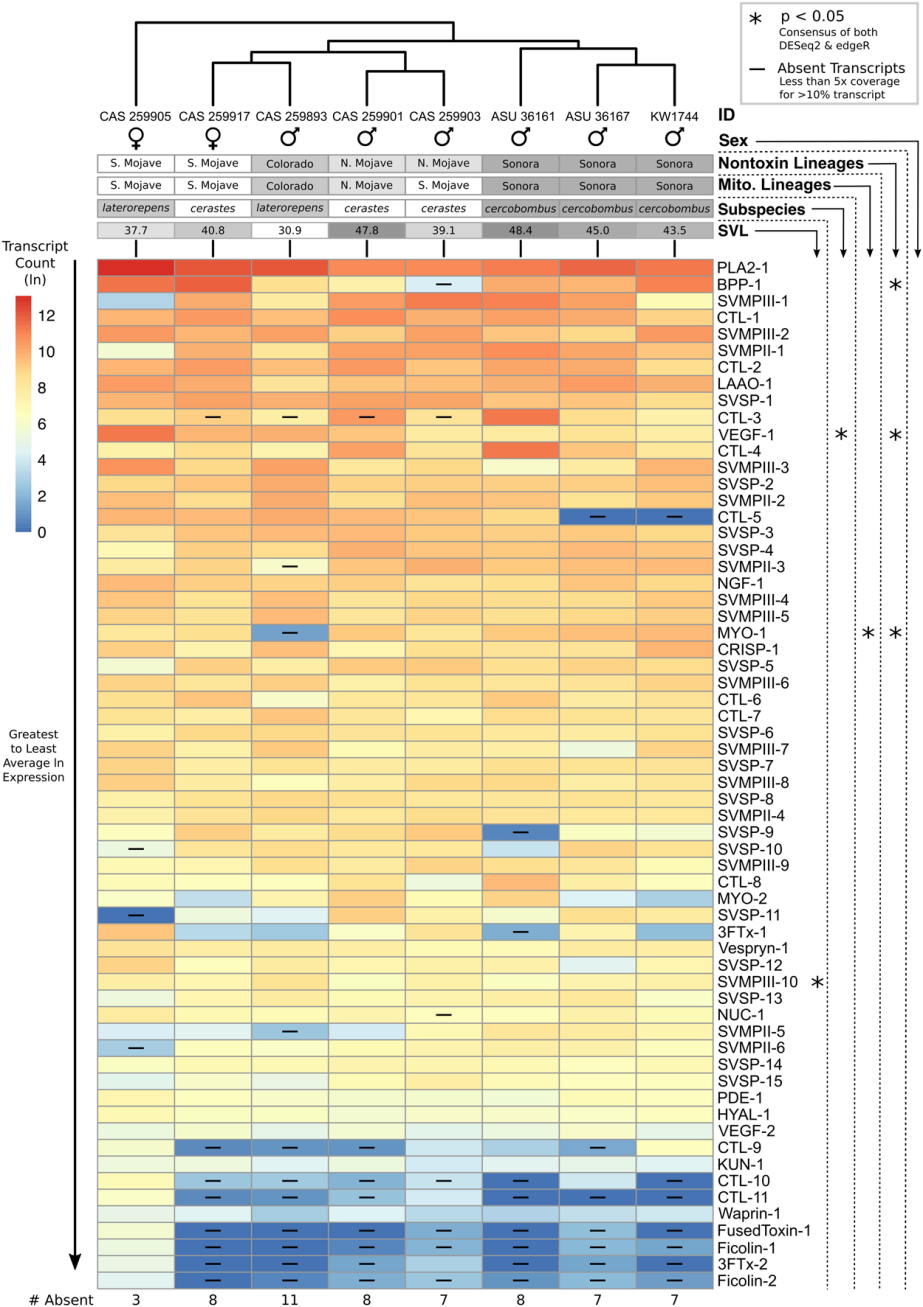
Hierarchical clustering of *Crotalus cerastes* expression similarity based on individual natural log-transformed toxin transcript TPM. Heatmap of toxins is ordered from greatest to least average ln expression with warmer colors indicating higher expression. Toxins with more than 10% of the coding sequence containing less than 5x coverage are marked as absent in that individual’s transcriptome. Detected significance for each transcript, based on consensus DESeq and edgeR analysis, is indicated with asterisks. Significant comparisons displayed in Fig. 4. SVL is reported in centimeters.

In contrast to our original hypothesis, similar toxin gene composition was found across all eight sampled individuals; however, differences in the presence or absence of toxins genes were recovered, providing some support for apparent variation in Sidewinder venoms as previously reported. While most toxins displayed similar expression across all individuals, 19 of the 62 toxin transcripts (31%) were absent in at least one individual, and none of the eight individuals contained all 62 transcripts (Fig. 3). Individual transcriptomes were missing between three and eleven toxin transcripts (4.8–17.7%), with an average of 7.4 (12%) missing. The majority of missing toxins were in lowly-expressed genes, though some highly-expressed toxins, such as CTL-3, were missing from one or more individuals (Fig. 3).

**Figure 4:**
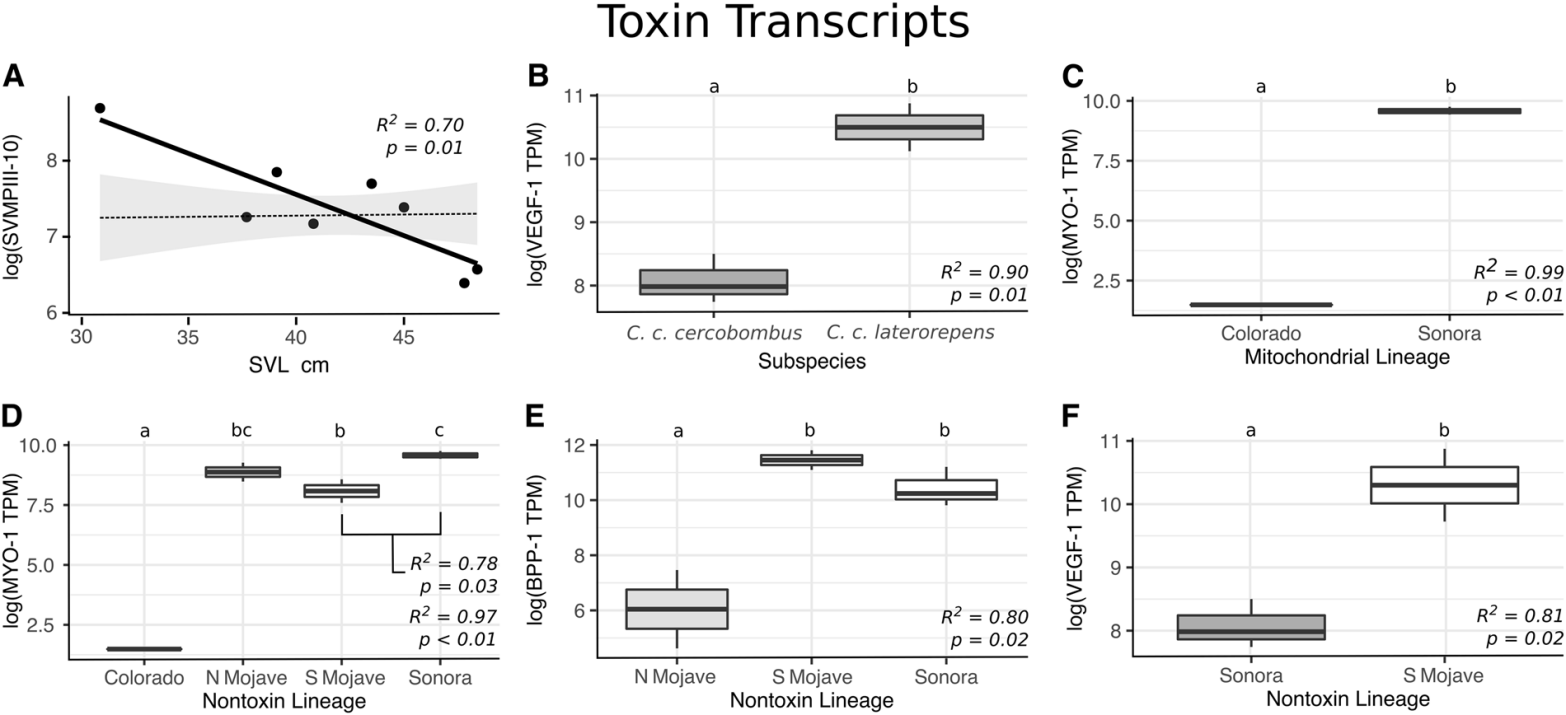
Significant relationships recovered by the consensus of DESeq2 and edgeR seen in Fig. 3. (**A**) SVMPIII-10 expression changes with size. Dotted-line and standard error represent the average toxin trend. (**B**) Expression of VEGF-1 in *C. c. cercobombus* relative to *C. c. laterorepens*. (**C**) MYO-1 expression in the mitochondrial Colorado lineage relative to the Sonoran lineage. (**D**) MYO-1 expression in the nontoxin Colorado lineage relative to all three of the remaining lineages; additionally, the Sonoran lineage maintained significantly higher levels of MYO-1 compared to the South Mojave lineage. (**E**) BPP-1 expression in the nontoxin North Mojave lineage relative to both the South Mojave and Sonoran lineages; given only one BPP was recovered, this relationship was retained in toxin class expression analyses in Fig. 5. (**F**) VEGF-1 expression in the Sonoran lineage relative to the South Mojave lineage.

**Figure 5:**
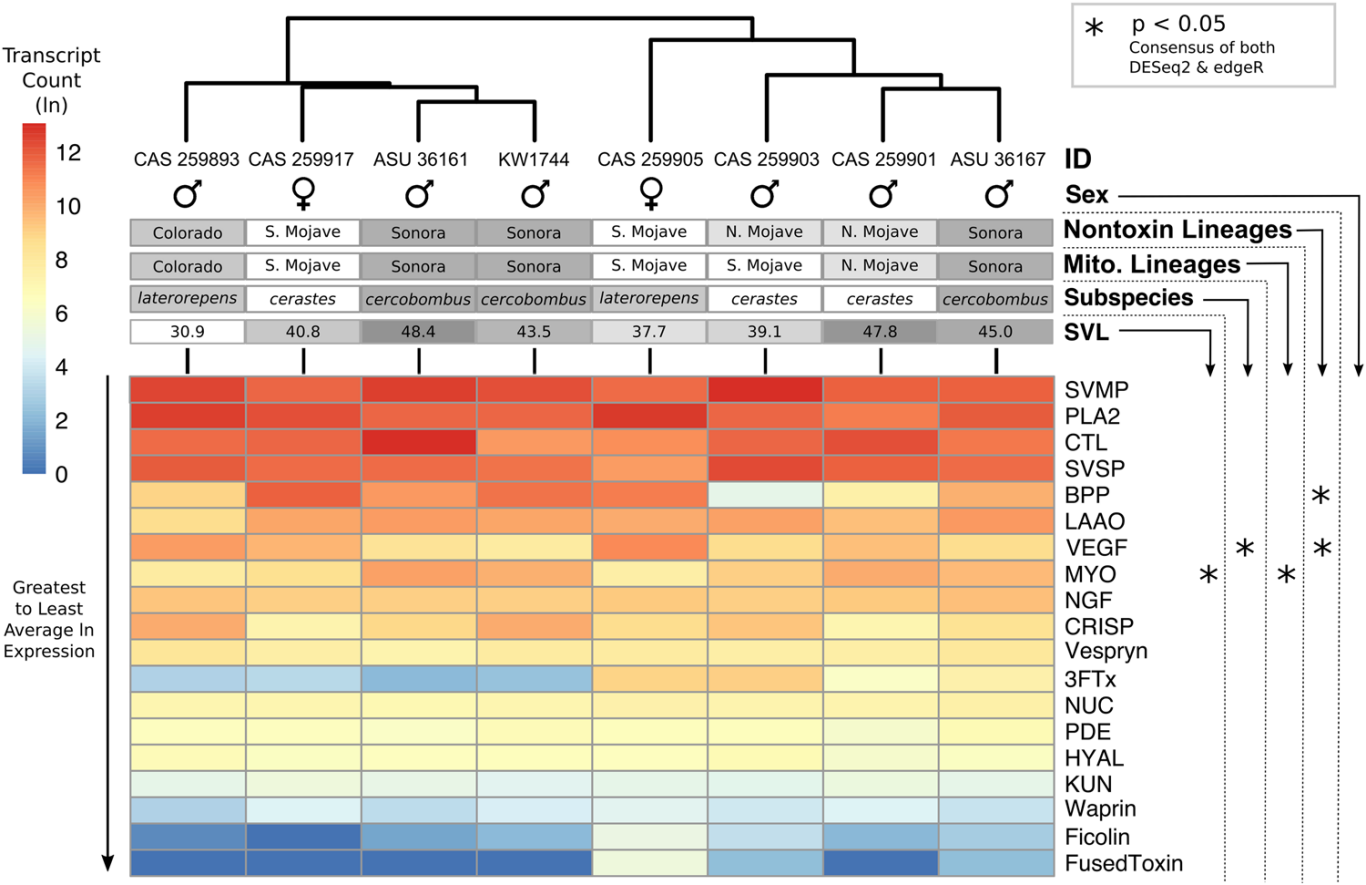
Hierarchical clustering of *Crotalus cerastes* expression similarity based on summed and natural log-transformed toxin classes. Heatmap of toxins is ordered from greatest to least average ln expression with warmer colors indicating higher expression. Detected significance for each transcript, based on consensus DESeq and edgeR analysis, is indicated with asterisks. Significant comparisons displayed in Fig. 6. SVL is reported in centimeters.

**Figure 6:**
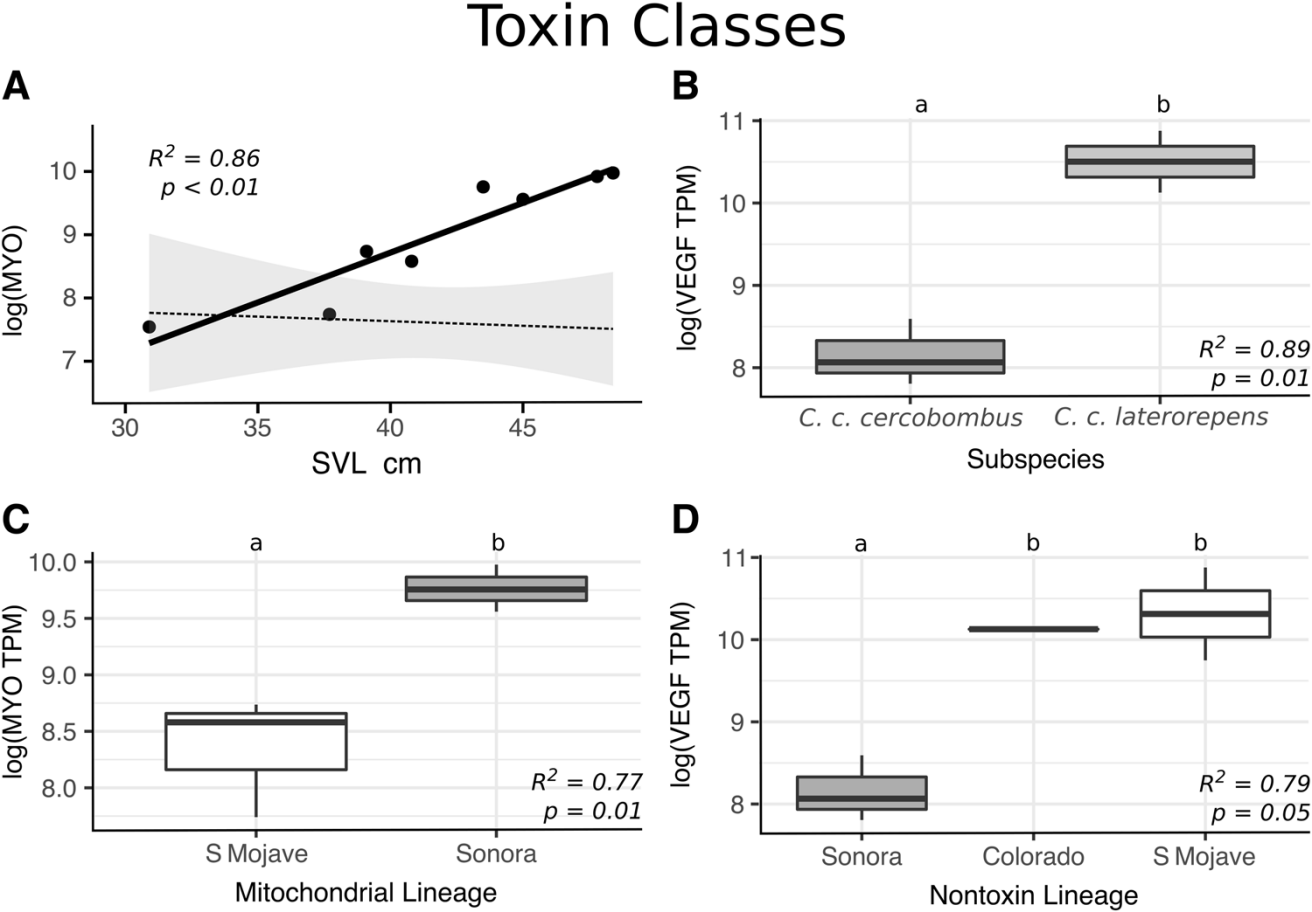
Significant relationships recovered by the consensus of DESeq2 and edgeR seen in Fig. 5. (**A**) Expression of combined myotoxins (n = 2) with size. Dotted-line and standard error represent the average toxin trend. (**B**) Expression of combined VEGFs (n = 2) in *C. c. cercobombus* relative to *C. c. laterorepens*. (**C**) Expression of combined myotoxins (n = 2) in the mitochondrial South Mojave lineage relative to the Sonoran lineage. (**D**) Expression of combined VEGFs (n = 2) in the nontoxin Sonoran lineage relative to both the South Mojave lineage and Colorado lineage.

Unexpectedly, few toxin transcripts were differentially expressed across the majority of tested factor comparisons, contradicting our earlier prediction that Sidewinder venom variation would correspond to life history or lineage assignment. Overall, only four of 62 toxin transcripts (10 of 1,054 comparisons) and three of 19 toxin classes (7 of 323 comparisons) were recovered as differentially expressed across any tested factors (Fig. 3; Supplementary Table S1). Only three cases of differential expression (consisting of three toxin transcripts) were recovered across 682 comparisons of life stage (Figs 3 and 4A), sex (no difference), subspecies (Figs 3 and 4B), and mitochondrial lineage assignment (Figs 3 and 4C). Similarly, expression of each toxin class lacked major patterns of differential expression across these factors (Fig. 5; Supplementary Table S2), as only two of 19 toxin classes (3 of 209 comparisons) were recovered as differentially expressed across life stage (Figs 5 and 6A), sex (no differences), subspecies (Figs 5 and 6B), and mitochondrial lineage comparisons (Figs 5 and 6C; Supplementary Table S1).

Notably, despite few instances of differential expression overall, more instances of expression differences were recovered between comparisons of nontoxin (phylotranscriptomic) lineages than all other comparisons combined. While only one individual changed lineage assignment using the more robust dataset, the number of significant results more than doubled. Seven of the remaining 372 comparisons for individual transcripts–consisting of a total of three toxin transcripts–were differentially expressed (Figs 3 and 4D–F); Supplementary Table S1). The number of significant toxin class comparisons also increased: VEGFs and BPPs were recovered as differentially expressed–four of the remaining 114 comparisons for toxin classes (Figs 5 and 6D; Supplementary Table S2). Given that only one BPP is present in the venom of *Crotalus cerastes*, this result reflected those of independent transcript analysis (Fig. 4E). This pattern of increased differential expression was mirrored with nontoxin transcripts as only 32 of 21,747 comparisons were found to be significant across all comparisons of life stage, sex, subspecies, and mitochodrial lineage assignments (Supplementary Table S1). However, across all comparisons of nontoxin lineage assignments, 55 of 11,862 comparisons were found to be significantly differentially expressed (Supplementary Table S1). In addition to the lack of significant differential expression, we detected significant phylogenetic signal in only one transcript, CTL-5 (*K* = 1.35; *P* = 0.02; Supplementary Table S3). As *K* was greater than one, it suggests the similarity in the expression of this toxin between closely related individuals was higher than expected under Brownian motion. This may have been driven by the absence of CTL-5 in the two most closely related Sonoran lineage samples (ASU 36167 and KW1744; Fig. 3), as the toxin was moderately to highly expressed in all other individuals.

### Venom proteomics and transcript versus protein abundance estimates

**Figure 7:**
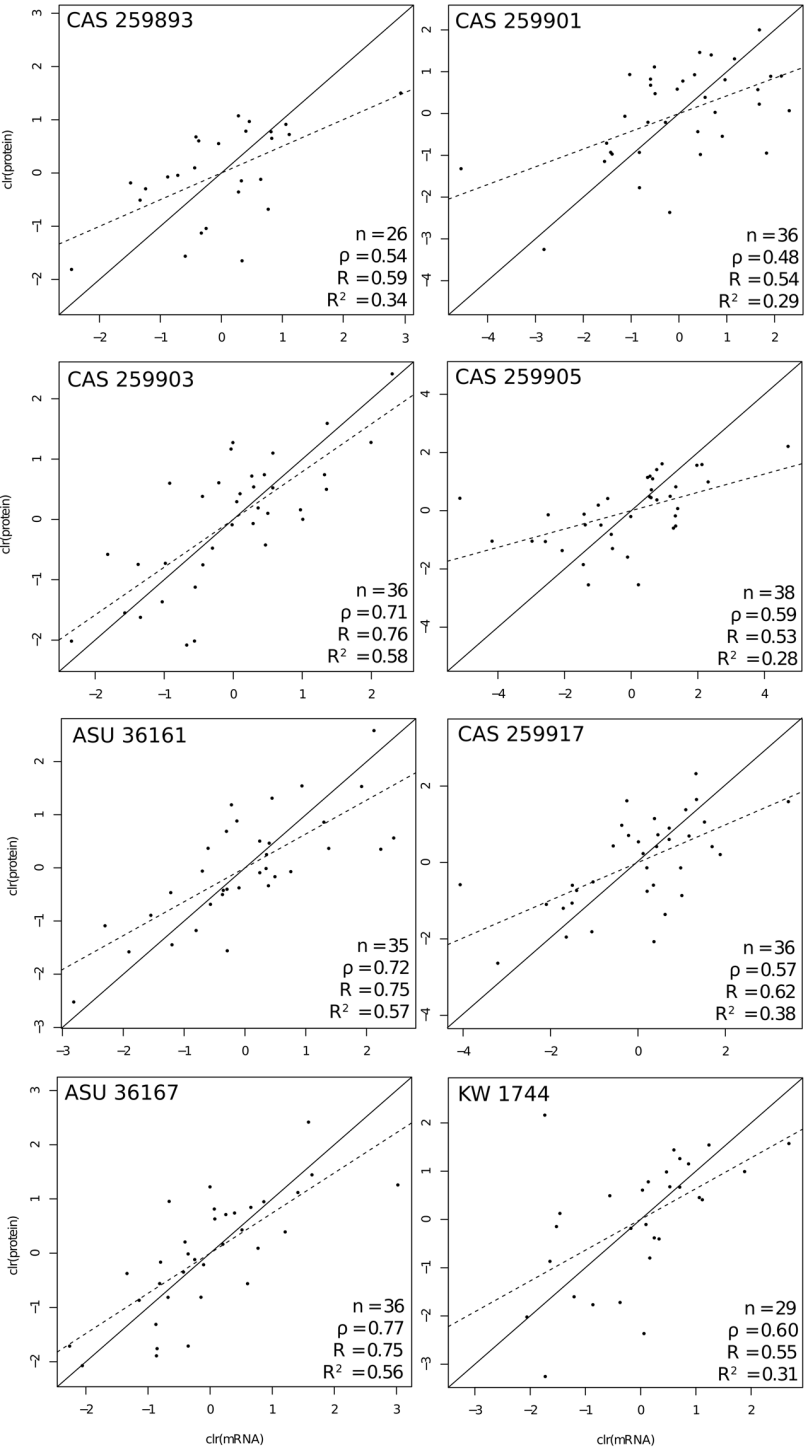
Correlation of venom transcript and protein abundances for eight *Crotalus cerastes*, showing positive correlation in all individuals. Data were centered log-ratio transformed (clr). Abbreviations: n - number of transcripts; *ρ* (Spearman’s rank correlation coefficient; *R* - Pearson’s correlation coefficient; *R*^2^ - coefficient of determination.

The majority of toxin transcripts were verified proteomically, and the unverified toxins were nearly all the lowest expressed across all individuals. With qMS, 49 of the 62 (79%) toxin transcripts were found in at least one individual; of these, 26 (53%) were conserved across all individuals, and nine additional transcripts were missing from two or fewer individuals. Toxins that were not verified in any individual were largely those most lowly expressed, and included all 3FTx, Ficolin, FusedToxin, KUN, and Waprin transcripts, as well as VEGF-2 and several CTLs (9, 10, and 11) (Fig. 2). Only CTL-4 was highly expressed but not verified proteomically at a 1% FDR; this transcript was recovered in four individuals at less stringent FDRs (2% in one individual, 10% in three others). Seven of the individual proteomes each contained 40–45 confirmed toxins; a juvenile—CAS 259893—had only 34; this was the smallest individual and similarly contained the highest number of suspected absent toxins in the transcriptome. Two of the most common toxin families, SVMPs and SVSPs, were conserved across nearly all individuals, as 24 of the 29 confirmed in those two families (83%) were found in all or all but one of the individuals. CTLs, on the other hand, were more variable: only two of seven confirmed (29%) were found across all individuals. Comparisons of transcriptomic and proteomic abundances were positively correlated, though the strength of the correlation was weaker in some individuals compared to others (Spearman’s rank correlation coefficients: 0.48 ≤ *ρ* ≤ 0.77; Pearson’s correlation coefficients: 0.53 ≤ *R* ≤ 0.76; coefficients of determination: 0.28 ≤ *R*^2^ ≤ 0.58; Fig. 7). As protein identity was based on the species-consensus transcriptome rather than individual transcriptomes, such differences in correlation were expected. Rokyta *et al*.^49^ found that venom-gland transcriptomes and venom proteomes were highly correlated in snakes (0.47 ≤ *ρ* ≤ 0.89; 0.58 ≤ *R* ≤ 0.92; 0.34 ≤ *R*^2^ ≤ 0.85), and the majority of our relationships were similarly correlated.

## Discussion

In this study, we first characterized venom-gland transcriptomes together with complementary venom proteomes of Sidewinder Rattlesnakes (*Crotalus cerastes*) for the first time. We also tested for differential expression across hypothesized lineages, subspecies, and life history traits to understand the effect of shallow lineage divergence on venom. Similar to other rattlesnakes, we found that *C. cerastes* venom largely consisted of SVMPs, SVSPs, a PLA2, CTLs, and a BPP. The most highly expressed toxins in *C. cerastes* venom are primarily responsible for hemorrhagic activity and necrosis (SVMPs), as well as myotoxic activity and the inhibition of platelet aggregation (PLA2s)^10,72^. The abundance of SVMPs and presence of PLA2 supports previous reports of moderate hemorrhagic activity and low-to-moderate PLA2 activity in *C. cerastes* relative to other *Crotalus*^35^.

Variation in Sidewinder venom-gland transcriptomes was largely not attributable to hypothesized lineages, subspecies, or life history traits, as we had originally predicted. Similarly, we recovered only one instance of significant phylogenetic signal in venom gene expression across our phylogeny, which appeared to be driven by the absence of that toxin in two individual transcriptomes. In fact, most toxin transcripts were not differentially expressed in the majority of the comparisons tested. One exception were myotoxins, supporting previous work by Bober *et al*.^24^ that detected myotoxins in the venom of samples of *C. c. cerastes* from southwestern Utah, but not from pooled commercial samples of unknown origin. We found MYO-1 to be expressed significantly less in the nontoxin Colorado lineage than any other lineage. This difference appeared to be based on transcript presence/absence: the gene encoding MYO-1 may be absent from the genome of the Colorado lineage, or not expressed. It also may be the result of an ontogenetic change rather than lineage-specific adaptations, as the Colorado individual was the smallest juvenile, and myotoxin class expression increased with size. One additional transcript, SVMPIII-10, decreased expression with size. As *Crotalus cerastes* undergo a known ontogenetic dietary shift^40^, these shifts in venom expression suggest a possible trade-off between these toxins at different life stages.

The most notable factor influencing differential expression in our dataset was lineage assignment based on analysis of the large nontoxin dataset. This analysis only re-assigned CAS 259903 from the South Mojave to the North Mojave lineage, but this single change more than doubled the number of significantly differentially expressed comparisons of toxins and a similar increase was found when comparing nontoxin transcripts. Discordance among morphological (subspecies), mitochondrial, and genomic datasets is common in systematics and phylogeography, and can be caused by differences in rates of evolution, inheritance patterns, introgression, or sex-biased dispersal^29–34^. Here, we demonstrate that improper assignment of lineages can misinform conclusions regarding the differential expression of transcripts across a phylogeny. Therefore, robust phylogenomic datasets, such as the thousands of nontoxins already sequenced as “bycatch” during venom gland transcriptomic studies, should be considered when testing for differential expression between clades.

Unlike some rattlesnake species, including taxa co-distributed through much of their range, Sidewinder venom did not form multiple discrete phenotypes, or follow a geographic pattern of variation. Mojave Rattlesnakes (*C. scutulatus*), a species sympatric with *C. cerastes* in much of the southwestern United States, exhibits dramatic cases of venom differentiation with hemorrhagic and neurotoxic phenotypes in short geographic distance, which seems to be driven by the presence or absence of neurotoxic PLA2 genes in their genomes^38,73–78^. Similar cases of dramatic venom differentiation at short geographic distance can be observed in Timber Rattlesnakes (*C. horridus*) and Southern Pacific Rattlesnakes (*C. helleri*)^36,37,45^. The lack of differential expression in Sidewinder venom is especially notable in light of a recent study examining intraspecific venom variation in two populations of the Common Lancehead (Viperidae: *Bothrops atrox*)^21^. Using similar methods to our own, 124 of 152 toxins (81.5%) were found to be differentially expressed between two genetically-distinct, allopatric populations^21^. In contrast, only four of the 62 (6.4%) toxins found in the Sidewinder venom-gland transcriptome were differentially expressed across four lineages. This lack of expression variation in Sidewinder venom between lineages and life history may be due to a variety of evolutionary processes, such as migration patterns or relaxed selection pressures.

While geographic structure of venom expression is well-documented in many species, examples of species lacking expression differences are known. For example, *Micrurus fulvius* populations across Florida were found to have a conserved venom phenotype^19^, which was hypothesized to be the result of either a species-wide selective sweep or a recent range expansion. Within Sidewinders, the lack of differential expression does not necessarily signify a conserved venom phenotype. While the expression of toxin classes, such as SVMPs, CTLs, and SVSPs, was largely similar across all individuals, the expression of individual toxin transcripts themselves was variable. The minimal differential expression in Sidewinder venom composition despite individual variation suggests that gene flow is likely occurring between the shallow lineages. Gene flow across the range of the species, as evidenced by the incongruence between our mitochondrial and nontoxin phylogenies, may have precluded local adaptation shaping one or more discrete venom phenotypes by swamping local adaptation to local prey abundance^79,80^. The individual variability could also be associated with a flat adaptive landscape or relaxed selective constraints on some more lowly-expressed toxins, allowing variation to arise through non-deleterious mutations over time^81^.

Further exploration of the drivers of venom variation in *Crotalus cerastes* should focus on sequence evolution rather than differential expression. While sequence evolution generally occurs more slowly than regulatory evolution, if Sidewinders are under relaxed selection or maintain high gene flow between lineages, these patterns would be apparent in the sequence data and could be explicitly tested. Most importantly, further studies involving venom variation of *C. cerastes* requires more thorough sampling, including the remaining south Sonoran mitochondrial lineage, and pairs of adults and juveniles within each lineage. As our sampling of juveniles was sparse, our conclusions regarding both ontogenetic shifts and pairwise lineage comparisons were limited. Similarly, our sample size was on the low end of what is required for detectable significance in tests of phylogenetic signal^69^; more comprehensive sampling would greatly increase the statistical power of such analyses.

In many snake venom-gland transcriptome studies, a single individual’s transcriptome is presented as characteristic of the entire species^44,82^. The differences in toxin gene composition across eight *Crotalus cerastes* transcriptomes reinforces the importance of using multiple samples to fully characterize the toxin arsenal of a species^20^; while a single sample will provide the majority of toxins for a species, it may lack some toxins that are present, even highly expressed, in others. In one case, the second most abundant toxin gene (BPP-1) was absent from the transcriptome of one individual (CAS 259903), and others were missing specific copies of other gene families (*e.g.* CTL-3 and CTL-5 missing from four and two individuals, respectively). This is especially true when *de novo* assembling and annotating transcriptomes, as 19 of 62 transcripts were missing from at least one individual. Without sampling the transcriptomes of multiple individuals and across the distribution, entire transcripts may be missed, and potential variation overlooked.

In conclusion, we provide the first venom-gland transcriptomes and complementary venom proteomes of *Crotalus cerastes* from individuals across their southwestern United States range. Our original hypothesis—that Sidewinder venom composition and gene expression is variable as a result of lineage diversification and traceable to specific life history or lineage assignments—was only partially supported, as individual variation in composition did not translate to extensive differential expression in the factors we tested. The relative lack of differential expression may be the result of high gene flow, relaxed selective pressures, individual stochasticity in venom overwhelming any potential signal from the factors tested, or local adaptation. Importantly, we found differences in the number of differentially expressed toxins between subspecies, mitochondrial lineage, and phylotranscriptomic lineage comparisons. Indeed, incorporating robust phylogenetic hypotheses, especially for taxa with poorly-explored evolutionary histories, is necessary for accurately detecting differential expression in transcriptomic studies relying on lineage assignments as hypotheses. Finally, studies looking to generate a species-consensus venom-gland transcriptome must sample multiple individuals in order to capture individual variation and the full venom repertoire of a species.

## Electronic supplementary material

Supplementary Table S1

Supplementary Table S2

Supplementary Table S3
